# Social Isolation, Loneliness, and Pace of Aging Among Older Adults in the English Longitudinal Study of Ageing

**DOI:** 10.1093/geroni/igaf122.4010

**Published:** 2025-12-31

**Authors:** Yifan Shi, Arun Balachandran, Heming Pei, Daniel Belsky

**Affiliations:** New York University, New York, New York, United States; Columbia University, New York City, New York, United States; UNC Gillings School of Global Public Health University of North Carolina at Chapel Hill, Chapel Hill, North Carolina, United States; Columbia University, New York, New York, United States

## Abstract

Loneliness and social isolation are prevalent among older adults and have been linked to increased morbidity and mortality. Emerging evidence suggests that social disconnection may also accelerate biological aging, yet few studies have examined the bidirectional relationship between psychosocial factors and aging processes using longitudinal data and validated biological aging measures. Using data from the English Longitudinal Study of Ageing, we investigated reciprocal associations between loneliness, social isolation, and the Pace of Aging (PoA), a biomarker-based measure of multisystem physiological decline. The analytic sample included 5,659 adults aged ≥50 with repeated blood biomarker, physical, and functional assessments across three timepoints. Loneliness and social isolation were measured at baseline and follow-up. Linear regression models were fitted, stratified by sex and age group, and adjusted for demographic, socioeconomic, health, and behavioral covariates. We found that baseline loneliness, but not social isolation, predicted faster PoA (β = 0.03), with stronger effects among men and adults aged younger than 70. In turn, faster PoA prospectively predicted higher levels of both loneliness (β = 0.12) and social isolation (β = 0.11) at follow-up, independent of baseline levels. These bidirectional associations persisted after adjustment, suggesting a reinforcing cycle between social and biological vulnerability. Findings highlight loneliness as a distinct and modifiable risk factor for accelerated aging and demonstrate the value of PoA as a responsive endpoint in social and behavioral intervention research. Addressing loneliness, particularly among men and the younger-old adults, may be critical for promoting healthy longevity and reducing disparities in aging outcomes.

